# Curvilinear MetaSurfaces for Surface Wave Manipulation

**DOI:** 10.1038/s41598-018-36451-8

**Published:** 2019-02-28

**Authors:** Luigi La Spada, Chris Spooner, Sajad Haq, Yang Hao

**Affiliations:** 1 School of Electronic Engineering and Computer Science Queen Mary University of London, London, E1 4NS, United Kingdom; 2 QinetiQ Ltd, Cody Technology Park Ively Road, Farnborough Hampshire, GU14 0LX United Kingdom; 3 School of Engineering and the Built Environment, Edinburgh Napier University, 10 Colinton Rd, Edinburgh, EH10 5DT United Kingdom

## Abstract

Artificial sheet materials, known as MetaSurfaces, have been applied to fully control both space and surface waves due to their exceptional abilities to dynamically tailor wave fronts and polarization states, while maintaining small footprints. However, previous and current designs and manufactured MetaSurfaces are limited to specific types of surfaces. There exists no general but rigorous design methodology for MetaSurfaces with generic curvature. The aim of this paper is to develop an analytical approach to characterize the wave behavior over arbitrary curvilinear MetaSurfaces. The proposed method allows us to fully characterize all propagating and evanescent wave modes from the MetaSurfaces. We will validate the proposed technique by designing, realizing and testing an ultrathin MetaSurface cloak for surface waves. Good results are obtained in terms of bandwidth, polarization independence and fabrication simplicity.

## Introduction

Recently, there have been increasingly renewed interests in the study of electromagnetics based on applications of metamaterials which possess extraordinary properties not existing in nature^1,2^. In addition to those conceptual designs such as perfect lens and invisibility cloaks, metamaterials have been used in manipulating electromagnetic waves by controlling their amplitude and phase ranging from microwaves to optics for engineering applications: telecommunications^3,4^, computing and data^5,6^, sensing and medicine^7,8^. Their two-dimensional analogues (MetaSurfaces) have found even greater potentials in the quest for modernizing microwave components and systems with small footprints and high frequency operations, as well as revolutionizing optical systems leading to so called “flat optics”^9^. MetaSurfaces are composed of arrays of metallic/dielectric inclusions, either periodically or randomly dispersed, whose dimensions and spatial periodicity are much smaller compared to the operative wavelength. As a result, MetaSurfaces shall exhibit unprecedented electromagnetic responses and material properties not found in materials existing in nature^10,11^. In particular:

- The possibility to arbitrarily manipulate their electromagnetic responses (i.e., resonances, levels of reflection/refraction/diffraction, phase distribution, impedance and polarization states) by simply changing physical dimensions, shapes, angular distributions of unit-cell inclusions.
- The possibility to be integrated with other materials such as semiconductors, piezo-electrics, ferro-magnetics and graphene for dynamic control of wave behavior from coherent light control, time-reversal to polariton symmetry breaking etc.

MetaSurfaces are typically implemented in the configuration of periodically array at microwave^12^, millimeter-wave^13^, and THz^14^ frequencies. By scaling dimensions of the unit-cell and modifying its material property, one can adapt the design into infrared region^15^. In addition to conventional design approaches traditionally used at RF and microwaves, novel techniques were recently introduced to design MetaSurfaces at optical frequencies such as Bouguer theorem^16^, carpet-cloak^17^ and metamaterials for surface plasmon manipulations^18–21^. Unfortunately, all such methods are valid only for specific shape, geometries, excitation source, and polarization of the impinging electromagnetic wave. Moreover, in practice, many apparatuses contain complex curvilinear surfaces which often scatter electromagnetic waves in an uncontrollable manner. To date, this is still an unsolved electromagnetic problem. Practically, until now, there have no generic and versatile design tools for implementing MetaSurfaces over arbitrary shape structures. To this regard, in this work, we will present a systematic approach for the design of curvilinear MetaSurfaces and the control of surface-waves propagation properties. The entire path will be defined, from specifications and structure design to its manufacturing.

The proposed approach consists of the following steps:

1. In the modeling section we obtain the mathematical description of the electromagnetic interaction between surface waves and the structure. Specifically, by using the field theory^22^, we relate the electric (**E**) and magnetic (**H**) field components with the structure constitutive parameters (electric permittivity ε(**r**) and magnetic permeability µ(**r**)). Such relation is contained in the Impedance *Z*(**r**), function of both electric (E(**r**)) and magnetic (H(**r**)) field components.
2. In the design section, we will find the MetaSurface physical characteristics. In other words, through the circuit theory^23^, we link the structure electromagnetic properties (**ε** and **µ**) with its geometry and dimensions. The Impedance *Z*(**r**), found in the modeling section, permits to obtain the lumped elements of the circuit model, namely: capacitance *C* and inductance *L*.
3. Finally, we will use the proposed approach to practically realize and manufacture a 3D curvilinear cloaking device for surface-waves. To develop the curved surface, we will use an appropriate geometrical transformation starting from the correspondent planar MetaSurface. The manufacturing technique follows the same methodological approach described in the design phase, namely. First, deposition of grid pattern on the flat dielectric substrate is carried out. Then, the curvilinear shape of the cloak is developed by using vacuum forming.

Sample measurements are performed using a near-field microwave scanning microscope to map surface wave distributions which are compared with those from analytical and numerical simulations.

The proposed study paves a new way for MetaSurface designs for different engineering applications ranging from microwave to higher frequencies.

## Materials and Methods

### Modeling: non-homogeneous Impedance

This section aims to obtain the Impedance *Z*(**r**) distribution over a curvilinear surface by solving Maxwell’s equations, according to the design specifications. Practically, this step links the structure electromagnetic characteristics with the surface-wave propagation properties (amplitude and phase).

Maxwell’s and Helmholtz equations in homogeneous dielectrics (ε_r_, µ_r_) are easily solvable, and their solutions (electric **E** and magnetic **H** field components) are straightforward for almost all the geometries we can envision^24^. On the other hand, in non-homogeneous media (ε(**r**), µ(**r**)) such equations became more complex and they are not always solvable. Therefore, a closed formula for the general solution is not available in literature: only solutions for specific permittivity/permeability profiles have been given in^25^. Here we develop a simple generalized formula to overcome such an issue.

**Figure 1:**
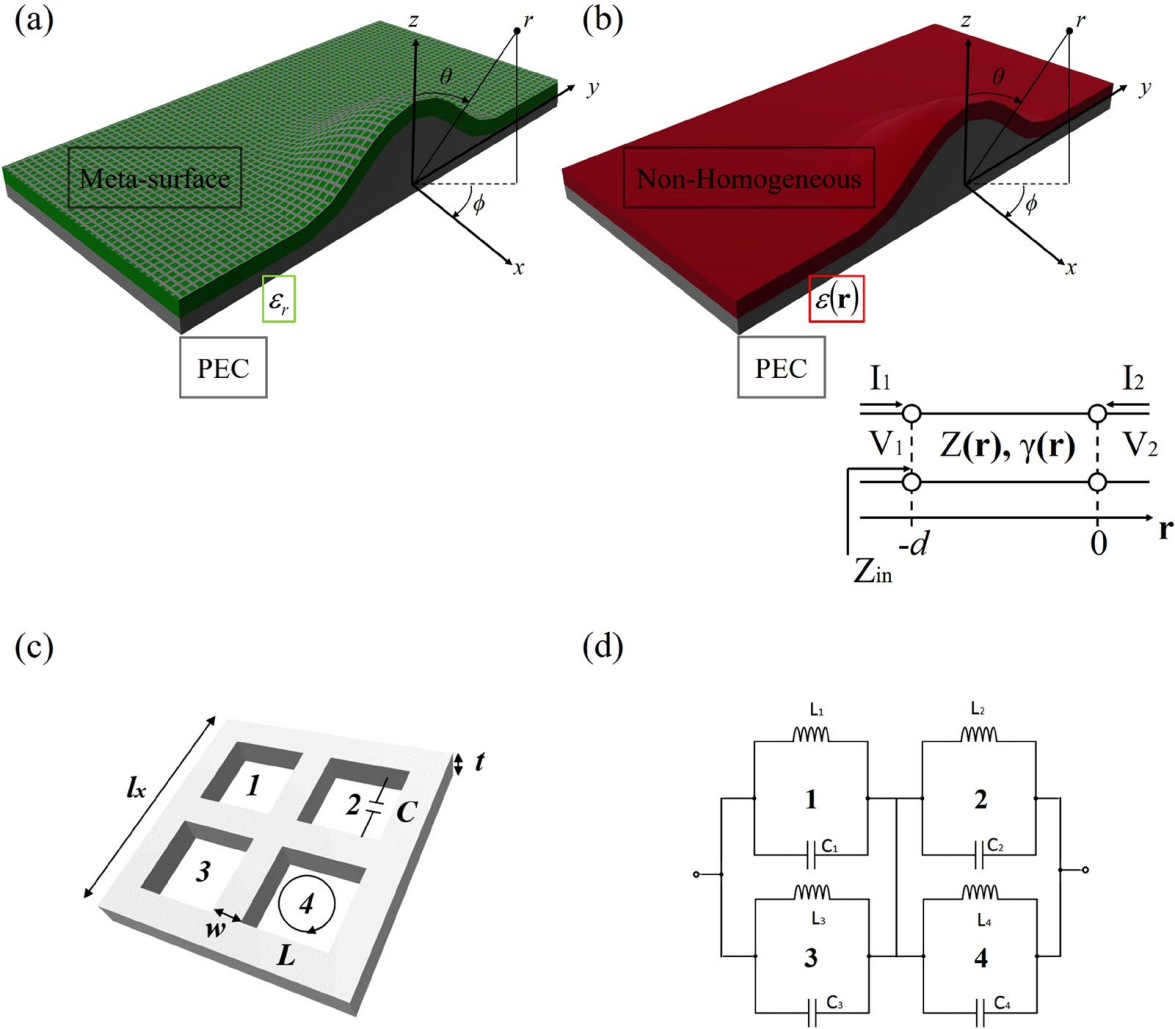
(**a**) Curvilinear MetaSurface device: metal ground plane (PEC), dielectric substrate (green), curved MetaSurface (grey); (**b**) Equivalent electromagnetic model: curvilinear non-homogeneous slab (red) in spherical coordinates; (**c**) MetaSurface unit-cell: *l* the square side length, *w* the strip width, *t* the metal thickness and *g* is the inner distance among the bar strips; (**d**) Equivalent circuit model: lumped elements geometrical capacitance and geometrical inductance.

Let’s consider the structure depicted in Fig. 1(a): a curvilinear grounded dielectric slab (with homogeneous permittivity ε_r_, green layer) on which a MetaSurface is deposited (grey grid). Differently to the existing works in literature, in this work we treat the MetaSurface as a real-life structure with its own thickness. Therefore, we can model it as a non-homogeneous layer with permittivity ε(**r**), function of the position vector r, as shown in Fig. 1(b). From^26^, both amplitude A(**r**) and phase Ф(**r**) for the electric **E** or magnetic **H** (=A(**r**)e^jΦ(r)^) components of the surface wave, can be related to the structure impedance *Z*(**r**) and electric permittivity ε(**r**), as follows:

1 $$\begin{array}{c}A({\bf{r}})=|\frac{Z({\bf{r}})-{Z}_{0}}{Z({\bf{r}})+{Z}_{0}}|\\ {\boldsymbol{\Phi }}({\bf{r}})={k}_{0}\int \sqrt{\varepsilon ({\bf{r}})}d{\bf{r}}+\phi \end{array}$$

Being *Z*_0_ the air impedance (*Z*_0_ = 377 Ω), k_0_ = 2π/λ_0_ the free space wave-number and λ_0_ the free space wavelength (See Supplementary Information file for mathematical details and demonstration).

The possibility to simultaneously control both amplitude and phase, is crucial to have a complete picture on how waves propagate on the MetaSurface.

### MetaSurface Design: from planar to curvilinear Impedance

In the previous paragraph we have found out the Impedance *Z*(**r**) profile of a generic curvilinear MetaSurface structure. In this section, we will obtain the relation between the structure Impedance *Z*(**r**) and its physical dimensions. To do so, an equivalent (LC) circuit model will be derived under the quasi-static approximation approach^27,28^.

The structure electromagnetic response is described by lumped circuit elements, namely: capacitance *C* and inductance *L*.

In real-life scenarios we deal with complex curvilinear structures. To obtain the equivalent circuit model (and the related inclusions physical dimensions) for the curved surface, it is necessary to start from the correspondent circuit model of the flat surface, and then apply the proper coordinate transformation. The aim is, known the dimensions of the planar inclusions, to obtain the related dimensions for the curvilinear inclusions. The designed impedance will permit to obtain the dimensions of the curvilinear inclusions to fabricate, satisfying the Impedance *Z*(**r**)/permittivity ε(**r**) distribution found in the modeling section.

The impedance is always a function of the geometry and the electromagnetic characteristics of the materials used. The evaluation of the resonant circuit impedance is related to the frequency range in which the structure operates. Typically, in the microwave region such terms depend exclusively on the size and geometry of the resonator. Consequently, such terms are labelled as “geometric capacitance” and “geometric inductance”^29^. However, at higher frequencies (e.g., THz, infrared, and visible), the thickness of the metal can no longer be neglected, and metals are not ideal conductors any more: additional effects arise and, in the equivalent circuit model of the inclusion, additional capacitance and inductance terms must be considered^30^.

The relationship between the planar Impedance and the circuit lumped elements for surface-waves reads^31^:

2 $${{\bf{Z}}}_{planar}(x,y,z)=\frac{j\omega {{\rm{L}}}_{planar}}{1-{\omega }^{2}{{\rm{L}}}_{planar}{{\rm{C}}}_{planar}}$$

where ***L*** and ***C*** represent the total inductance and total capacitance, respectively: such terms consider both the geometrical and (if any) the additional terms.

To conform the planar MetaSurface on the desired curved structure, we need to transform the planar Impedance **Z**_*planar*_ to the correspondent curvilinear one. To this regard, let’s consider a generalized orthogonal reference system, described by its orthogonal unit vectors (***q***_1_, ***q***_2_, ***q***_3_), spatial coordinates (*q*_1_, *q*_2_, *q*_3_) and metric factors (*h*_1_, *h*_2_, *h*_3_)^32^. Let’s suppose to increment one of the generic curvilinear coordinate *q*_1_ of a quantity d*q*_1_. The point P will move along the line *q*_1_, of a quantity d*s*_1_. Differently from the planar case, such quantity d*s*_1_ will not be equal to d*q*_1_, but a function of the considered shape, described by its metric coefficient *h*_1_. Similar considerations are valid for the other two metric coefficients *h*_2_ and *h*_3_^33^. Therefore, starting from the expression of the planar Impedance **Z**_*planar*_, we can derive the equivalent curvilinear Impedance **Z**(**r**), by using the appropriate coordinate transformation **α**(*h*_1_, *h*_2_, *h*_3_):

3 $$\mathop{\underbrace{[\begin{array}{c}{Z}_{q1}\\ {Z}_{q2}\\ {Z}_{q3}\end{array}]}}\limits_{{\bf{Z}}({\bf{r}})}=\mathop{\underbrace{[\begin{array}{ccc}\frac{{h}_{2}{h}_{3}}{{h}_{1}} & {h}_{3} & {h}_{3}\\ {h}_{3} & \frac{{h}_{1}{h}_{3}}{{h}_{2}} & {h}_{1}\\ {h}_{2} & {h}_{1} & \frac{{h}_{1}{h}_{2}}{{h}_{3}}\end{array}]}}\limits_{{\boldsymbol{\alpha }}({h}_{1},{h}_{2},{h}_{3})}\,\,\mathop{\underbrace{[\begin{array}{c}{Z}_{x}\\ {Z}_{y}\\ {Z}_{z}\end{array}]}}\limits_{{{\bf{Z}}}_{planar}(x,y,z)}$$

**Z**(**r**) is the vector containing the Impedances along the curvilinear coordinates (*q*_1_*, q*_2_*, q*_3_), **α** is the transformation matrix related to the curvilinear reference system adopted (*h*_1_, *h*_2_, *h*_3_), and **Z**_**planar**_ the vector containing the planar Impedances in Cartesian coordinates (*x, y z*).

In this way, we can link both surface-wave amplitude A(**r**) and phase Ф(**r**), with the curvilinear MetaSurface physical dimensions contained in **Z**(**r**). The proposed approach presents three main advantages:

1. Both electric and magnetic fields can be factorized to properly *model* any curvilinear interface in terms of Impedance ***Z***(**r**) and permittivity ε(**r**), reducing complexity in the analytical calculations and numerical simulations.
2. Suitable curvilinear reference systems can be synthesized (through **α**(*h*_1_, *h*_2_, *h*_3_)) as a function of the application required, to *design* a wide number of MetaSurface inclusion geometries;
3. By realizing the planar MetaSurface first, then conforming it to the target curvilinear shape, it is possible to simplify the *manufacturing* processes and lower the costs.

## Results and Discussion

### Curvilinear MetaSurface modelling

We now demonstrate an application of the proposed approach for surface-wave invisibility cloak. It is known that electromagnetic surface waves are strictly confined to the interface, traveling in a direction parallel to the interface itself, meanwhile its amplitude decreases with the distance^34^. Surface waves exist in a variety of structures involving different materials^35,36^, and can be excited by using different ways^37,38^.

Figure 1(a,b) show the geometry of the 3D curved metallic surface to cloak and its equivalent model, respectively. The top layer is air with dielectric permittivity ε_0_ and magnetic permeability µ_0_. The dielectric slab has thickness *h*, relative permittivity ε_1_ = ε_0_ε(**r**) and magnetic permeability µ_1_ = µ_0_, with r the position vector described in the spherical coordinate system (*r, θ, ϕ*). An impinging electromagnetic wave can assume different forms at the interface: reflected, transmitted and/or absorbed. Any alteration to the boundary conditions causes changes in the wave propagation characteristics in terms of amplitude and phase. Such modifications are mainly due to the angle of incidence θ_i_ and the critical angle θ_c_ of the considered interface. An electromagnetic wave that is properly fed into the device, can propagate and support the following modes^39^:

1. It can propagate into both air and slab, we refer to such modes as *air* radiated wave and *slab* guided modes.
2. It can be bounded and guided within the MetaSurface *substrate*, referred to as the *wave-guide substrate* modes.
3. It can propagate along both upper and bottom interfaces, referred as *surface wave* modes. They can be evanescent or slow waves, as a function of the value of the incident angle, compared to the related critical angles at the boundaries.

Even though here we are interested in surface-wave cloaking applications, it must be pointed out that the underlying theory can be easily applied to the other above-mentioned wave phenomena: radiated waves and wave-guide modes.

The aim of invisibility cloak devices is to let the waves propagate undisturbed along the structure, to render the object underneath invisible to the impinging electromagnetic wave. When cloaks are implemented using MetaSurfaces, the device is expected to *maintain* both amplitude and phase of the propagating surface wave unchanged before and after the object. From Fig. 1(b) it is clear that there are three distinct regions, dielectric *slab*, the MetaSurface and its substrate (represented as an *non-homogeneous* red layer), and *air*. As previously mentioned, both electric **E** and magnetic **H** field components must satisfy the non-homogeneous differential equation^32^ in spherical coordinates (*r, θ, ϕ*), by using the following distribution along the radial *r*, angular *θ*, and azimuthal *ϕ* directions, respectively:

4 $${{\boldsymbol{\varepsilon }}}_{profile}(r,\theta ,\varphi )=[\begin{array}{c}1-(\frac{2}{\pi }{r}^{2})\\ \frac{1}{{\sin }^{2}(\theta )}\\ {\varepsilon }_{r}\end{array}]$$

### Curvilinear MetaSurface design

To satisfy the profile (4), we first evaluate the equivalent planar impedance of the structure. Let us consider the inclusion geometry (cross-square shape) shown in Fig. 1(c) and its equivalent circuit model representation of Fig. 1(d). By using equation (2) and the proper boundary conditions, we obtain for the considered unit-cell:

5 $${Z}_{x}={Z}_{y}=\frac{j\omega 4({L}_{self}-M)}{1-{\omega }^{2}16({L}_{self}-M)({C}_{fring}+{C}_{surf})}$$

To determine the link between the Impedance and the physical dimensions for the planar case, we need to explicit all the inductive (*L*_*self*_ and *M*) and capacitive (*C*_*fring*_ and *C*_*surf*_) terms. The geometrical inductance can be easily obtained as the sum between the squared loop (*L*_*self*_) self-inductance^40^ and the mutual inductances (*M*) among the parallel bars^41^ as ${L}_{tot}={L}_{self}+M$. It reads:

61 $${L}_{tot}(l,w,g,t)=\mathop{\underbrace{{\mu }_{0}l(\frac{1}{2}-\frac{1}{5}\,\mathrm{log}(w/t))}}\limits_{{L}_{self}(l,w,t)}+\mathop{\underbrace{{\mu }_{0}\frac{1}{4\pi }[2l{\sinh }^{-1}(l/g)+2(l-w-\sqrt{{g}^{2}+{l}^{2}})]}}\limits_{M(l,w,g)}$$

The total capacitance can be expressed as the sum of the fringing capacitance considering the contribution of the non-parallel electric field lines of the non-adjacent metal plates (horizontal and vertical bars), *C*_*fring*_; and the surface capacitance due to the charges on the metallic surfaces *C*_*surf*_ as^42^ ${C}_{tot}={C}_{fring}+{C}_{surf}$. It reads:

6.2 $${C}_{tot}(l,w,g,t)=\mathop{\underbrace{{\varepsilon }_{0}{\varepsilon }_{r}\frac{2w+\sqrt{2}g}{\pi }{\cosh }^{-1}(\frac{2w+g}{g})}}\limits_{{C}_{fring}(w,g)}+\mathop{\underbrace{2{\varepsilon }_{0}{\varepsilon }_{r}\frac{t+w}{\pi }\,\mathrm{log}(\frac{8l}{\pi g})}}\limits_{{C}_{surf}(l,w,g,t)}$$

In parentheses, we explicitly show the dependencies on the geometrical and electrical parameters characterizing the inclusion (*l, w, g* and *t*) and the substrate (*ε*_*r*_).

To obtain the curvilinear Impedance, starting from the related planar Impedance, we need to apply equation (3) in the spherical coordinate case ${{\bf{Z}}}_{spherical}(r,\theta ,\varphi )={{\boldsymbol{\alpha }}}_{planar \mbox{-} spherical}{{\bf{Z}}}_{planar}(x,y,z)$, with the following matrix **α**_planar-spherical_^43^:

63 $${{\boldsymbol{\alpha }}}_{planar \mbox{-} spherical}=(\begin{array}{ccc}{r}^{2}\,\sin (\theta ) & r\,\sin (\theta ) & r\,\sin (\theta )\\ r\,\sin (\theta ) & \sin (\theta ) & 1\\ r & 1 & \frac{r}{\sin (\theta )}\end{array})$$

***Z***_spherical_ contains the dimensions of the unit-cell for the curvilinear MetaSurface. These are the dimensions necessary to satisfy the profile in (4) and cloak the metallic object.

### Curvilinear MetaSurface manufacturing

**Figure 2:**
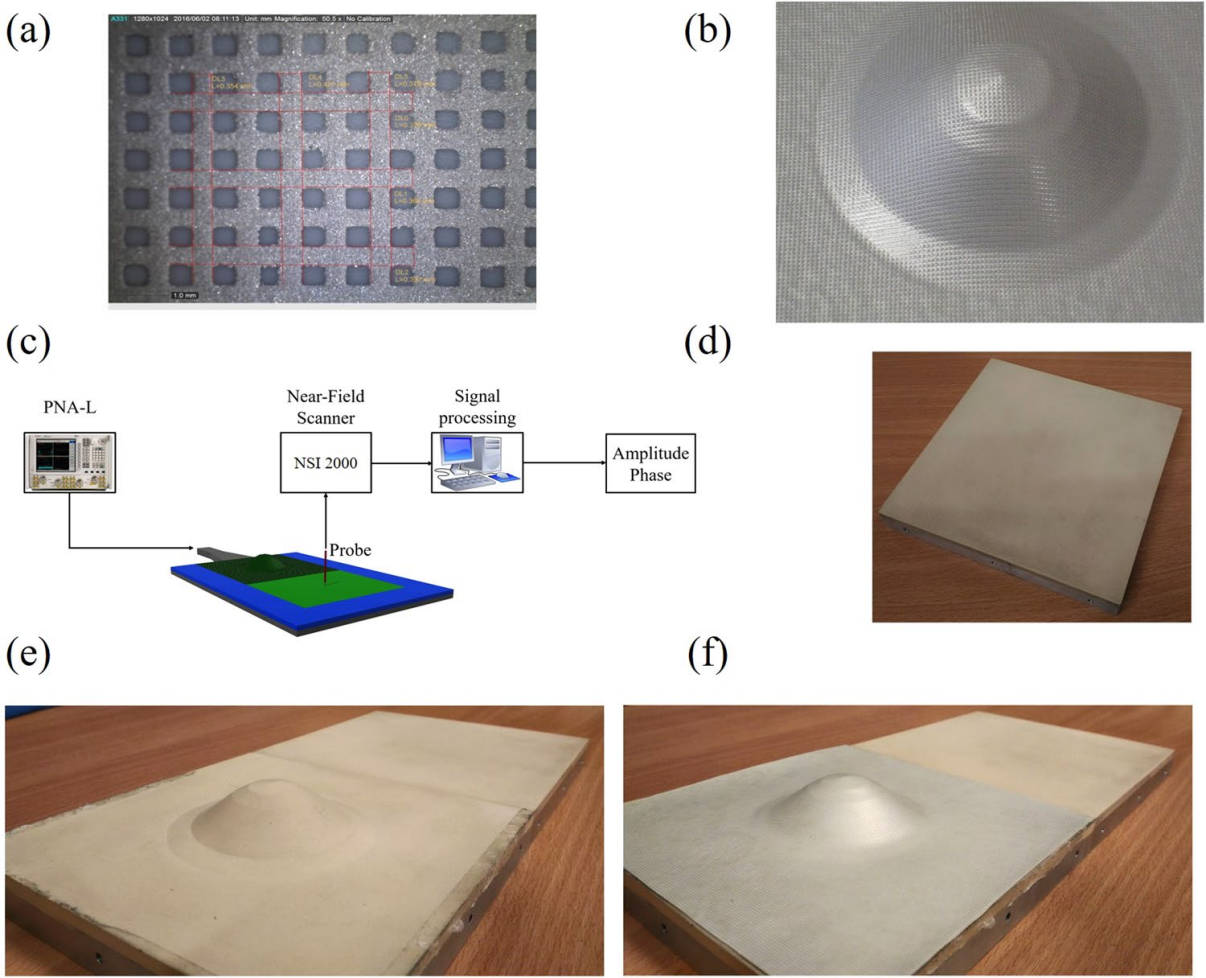
Manufacturing approach (**a**) step 1: deposition of a grid pattern on a dielectric substrate, (**b**) step 2: vacuum forming of the shape; (**c**) experimental setup used; Samples manufactured: (**d**) flat plane, (**e**) object with uniform dielectric layer, namely no-cloak, (**f**) object with MetaSurface on top, namely cloaking device.

The structure designed in the previous paragraph is synthesized by a non-homogeneous metallic grid, stacked on a dielectric supporting substrate. Its manufacturing process, shown in Fig. 2 can be broken down into two distinct stages: deposition of grid pattern on dielectric substrate and vacuum forming of cloak shape.

First, the conductive grid pattern was deposited onto High Impact Polystyrene (HIPS) planar sheets of size 300 × 300 mm using screen printing (deposition process performed by Viprotech Vipromat Executive screen printer). A silver loaded ink was printed through a polyester screen mesh with a thread count of 79 threads per cm and a thread diameter of 48 μm (giving a theoretical ink volume of 26.9 cm^3^/m^2^), the printed pattern applied to the screen using photo-emulsion processes. The screen mesh is used to transfer the printed pattern to the substrate. A metal doctor blade (dragging bar) is moved across the screen to fill the open mesh apertures with ink. On the reverse stroke, a polyurethane tipped squeegee blade deforms the screen into contact with the substrate momentarily along a line of contact. This causes the ink to wet the substrate and be pulled out of the mesh apertures when the screen recoils after the blade has passed. After printing, samples were immediately dried in a box oven at 80 °C for 30 minutes to minimize bleed of the printed pattern (Fig. 2(a)).

Then, once the conductive grid had been deposited onto the planar substrate, the substrate was formed into the 3-dimensional required curvilinear surface (vacuum forming process realized by Formech 450). In this process the polymer sample to be formed is first heated to its softening temperature. In the case of the Formech machine this is accomplished using an infrared panel heater which is slid into position above the substrate. The mould is then pushed into contact with the substrate and a vacuum applied between the substrate and the mould. The resulting imbalance of air pressure on either side of the substrate results in it taking the shape of the mould. A dwell time (5 seconds) was found to impart the correct thermal energy into the HIPS to soften but not melt the substrate. A vacuum pressure of 0.85 bar was then used to form the substrate over the mould. A porous breather tissue was positioned around the base of the mould to prevent the substrate from forming a gas seal around the edge of it during forming – locking off the vacuum pressure and preventing any further deformation of the substrate into the shape of the mould (Fig. 2(b)).

Other works on surface-wave cloaks have used existing Transformation Optics (TO) designs for free-space cloaks, but these suffer the drawbacks of being anisotropic, electrically large, and/or only working for a single direction of incident wave. The manufacturing approach used here is different from the method existing in literature. TO-based techniques are usually expensive and restricted to a small area, and not easily scalable, especially for complex shapes. In this work, instead, we fabricated the samples by producing the mesh first, then formed the shape post mesh deposition, as dictated by the design.

### Experimental setup and measurements

We have seen that the application of the method, developed here and described in the previous paragraphs, allowed us to design and manufacture the curvilinear MetaSurface structure. In this paragraph we are going to experimentally verify the reliability of the proposed approach.

The excitation is provided by a pyramid horn antenna with a central operating frequency of 10 GHz, attached to port 1 of an Agilent N5230C PNA-L network analyzer. An absorbing layer with electrical properties εr = 3.8–j7.2 at 10.0 GHz is used to bound the entire structure in the xy-plane and avoid unwanted reflections (Fig. 2(c)). By following the procedure detailed in the modeling and design section we have manufactured the MetaSurface structure accordingly. The central operational frequency is f_0_ = 10 GHz and the related wavelength reads λ_0_ = 30 mm, therefore in relation to the unit-cell of Fig. 1(c) the geometrical parameters are: side length l = λ_0_/15, w = λ_0_/75 and t = λ_0_/30.

Since we are interested in what happened to the surface wave propagating after the object, an additional flat dielectric substrate, used as reference sample (Fig. 2(d)) is placed next to the object to cloak for both samples: the no-MS (Fig. 2(e), object without the MetaSurface) and the MS (Fig. 2(f), object covered by the designed MetaSurface). The reason for the attached flat dielectric slab is to ensure that a sufficient amount of space was available in the forward scattering region to clearly reveal the performance of the cloak.

To detect the surface wave along the device, the NSI 2000 planar scanner has been used with a monopole probe positioned 0.5 mm above the surface and connected to port 2 of the PNA-L. With this two-port set up, the S21 parameter is measured along the sample (with a resolution of 1 mm × 1 mm) from which the corresponding electric field component normal to the plane is detected in terms of amplitude and phase.

Once we get the measured data a similar spectral analysis conducted in^38^ has been used to decompose the complex signals into simpler parts by following 4 crucial steps: (1) Fourier Transform from spatial domain to frequency spatial domain (normalized k-vectors space ${k}_{n}={\sqrt{{\varepsilon }_{r}}/2\pi \lambda }_{0}$), which contains both space and surface waves components. (2) Identification of space-wave (k_n_ = 0.033 being ε_r_ = 1) and surface-wave (k_n_ = 0.12 being ε_r_ = 13.9) in the bi-dimensional spatial frequency spectrum. (3) Filtering and isolation of surface wave component to filter out the unwanted space wave. (4) Conversion in the spatial domain: an inverse transformation is applied to go back to the spatial domain.

The validation of the proposed approach is done by comparing the experimental results obtained in the previous paragraph with the analytical model developed by using Mathematica^44^, and the numerical simulation results from the commercial full-wave electromagnetic software (CST)^45^. We compared the electric field component E_z_ in terms of amplitude and phase for the central frequency f_0_ = 10 GHz (λ_0_ = 30 mm), as reported in the plots inserted in the Supplementary Information file.

### The surface-wave cloak device

**Figure 3:**
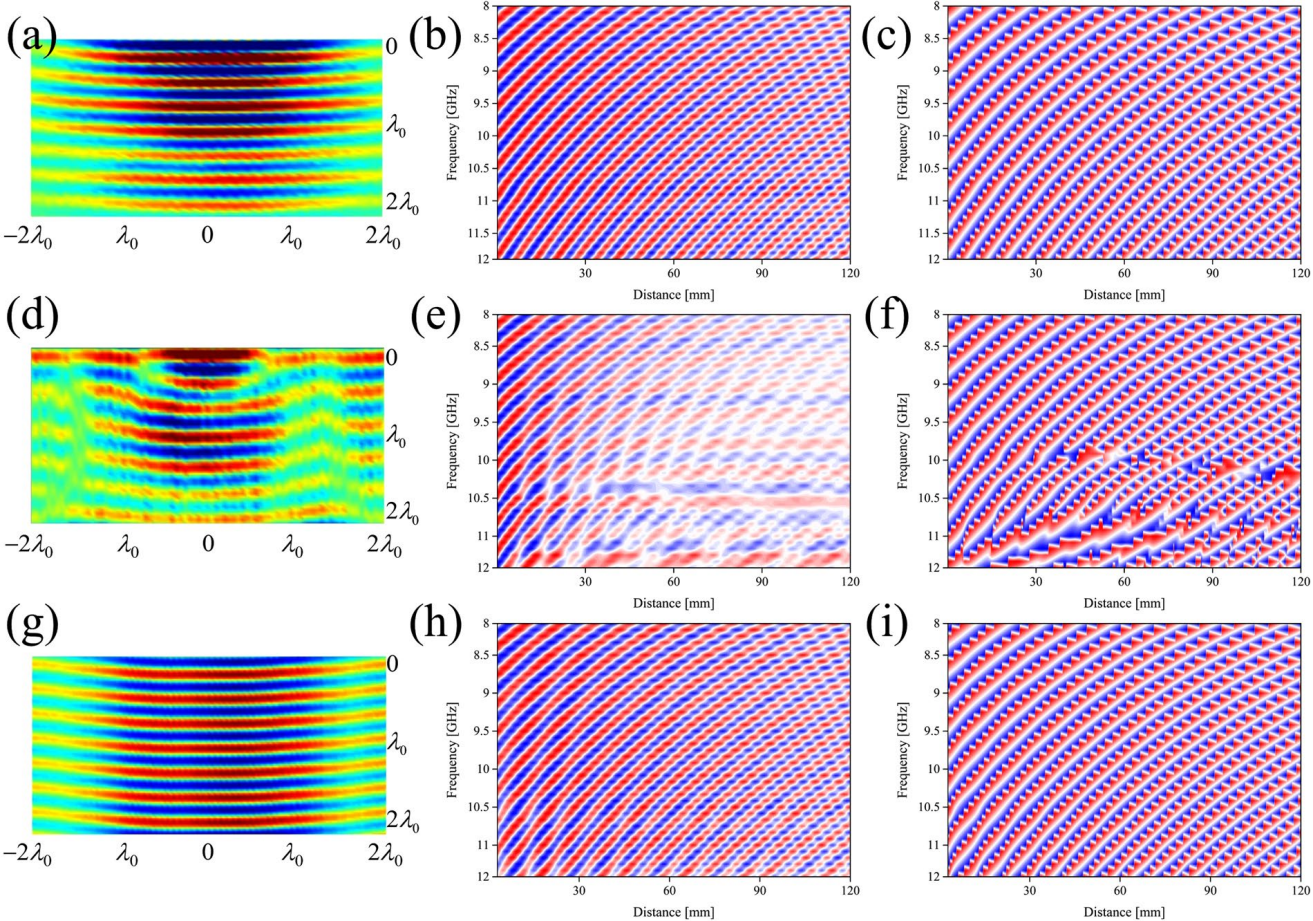
2D measurements electric field Ez component along x-y plane: (**a**) flat surface (reference), (**d**) object with uniform dielectric layer (no-cloak) and (**g**) object with meta-surface on top (cloaking device). Frequency considered f = 10 GHz. 2D frequency-distance plot for: reference amplitude (**b**) and phase (**c**); no-cloak amplitude (**e**) and phase (**f**); cloak amplitude (**h**) and phase (**i**).

With the setup described in the previous paragraph, we obtained the field distribution of the electric field that only contains the surface wave. The 2D measurements, for the samples: reference, object without the MetaSurface (no-MS) and object cloaked by using the designed MetaSurface (MS), are shown in Fig. 3.

In the flat dielectric slab with homogeneous permittivity (*reference*), the field is propagating undisturbed and possess the following configuration in spherical coordinates^46^: $E(r,\theta ,\varphi )={a}_{mn}{H}_{n}^{m}({\beta }_{d}r){P}_{n}^{m}(\cos \,\theta )\sin (m\varphi )$, where the radial variation *r* is represented by spherical Hankel functions for outwardly traveling waves. The azimuthal variations *θ* are described by Legendre functions P_n_^m^(cosθ). The angular variation *ϕ* can be written in the form of periodic functions such as cos(mϕ) or sin(mϕ) to describe even and odd modes, respectively. From the physical point of view, the energy confinement, crucial for cloaking applications, is high and the radiation from the surface is minimal at the central frequency f_0_ = 10 GHz (Fig. 3(a)) and as expected, also for the rest of frequencies in the range considered in terms of amplitude (Fig. 3(b)) and phase (Fig. 3(c)).

In the case of the curvilinear uniform dielectric slab (no-MS), we note a large amount of forward surface wave scattering due to the creation of interference patterns, Fig. 3(d). The presence of the object leads to a change in the wave incident angle θ_i_: the energy of the wave is not confined along the surface anymore, but it is transmitted in the upper and lower side of the structure: *radiated waves* and *wave-guide* modes, respectively. Due to the presence of both modes, the scattered amplitude (Fig. 3(e)) and phase (Fig. 3(f)) does not follow the pattern of the flat plane but decrease more rapidly compared to the reference case. This results in different interference patterns: the summation of all phases can be equal to zero (destructive interference) and/or the wave pattern will be a function of the position (non-uniform waves).

When the manufactured MetaSurface (MS sample) covers the metallic object, the impinging wave is perfectly reconstructed (Fig. 3(g)). The MetaSurface reduces the amount of both back and forward scattering, being accurate in the reconstruction of both wave amplitude and phase fronts for all the frequencies considered, Fig. 3(h,i), respectively. To better understand thhis operating principle of the cloaking device, it is necessary to start from how the structure is composed. The unit cell consists of one layer of metal square-shape particle on the top and its inverted dielectric pattern at the bottom, separated by conformal dielectric substrate: *l* and *w* are the length and width, respectively, *t* the thickness and *d* the space between the top and bottom layers.

In^47^ it has been demonstrated for radiated waves that the enhancement in the MetaSurface characteristics (bandwidth, insensibility to impinging polarization and losses) can be explained by considering two crucial electromagnetic phenomena: symmetry-breaking and intra-layer (among adjacent unit-cells) couplings phenomena.

As known in literature, single-layer metallic MetaSurfaces^48^ and their inverted version^49^ can only exhibits narrowband and limited radiation efficiency^50^. In literature it has been also demonstrated how breaking the symmetry of a structure can lead to a wideband behavior^51^ and polarization independency^52^. Therefore, by exploiting such a phenomenon we realized a bi-layer structure where its symmetry is broken thanks to the presence of metallic on top and its dielectric counterpart at the bottom. According to the dual principle^53^, if the structure has an E-field resonance mode, the complementary structure will exhibit a corresponding H-field resonance mode at the same frequency. If the E-field distribution on the top layer has an antisymmetric resonance, the H-field at the bottom dual structure will be symmetric, and vice-versa. Moreover, according to the surface equivalence theorem^54^, small apertures in a metallic screen, as in our case, can be treated as arrays of magnetic current elements: if **E**_**a**_ is the electric field on an aperture, it is equivalent to a transverse magnetic current J_**m**_ = − **n** × **E**_**a**_ on the aperture plane. The transverse electric current **J**_**s**_ flows, with high intensity along the metallic square-shaped particle, whereas the transverse magnetic current **J**_**m**_ is very weak in the same region, as expected. Dual results for the bottom complementary structure.

In previous realizations for single-layer MetaSurfaces, it has been assumed that the intra-layer coupling between adjacent unit-cells is negligible^55^. On the contrary, for our structure (and its cloaking purpose) such an effect cannot be neglected, it plays a crucial role in controlling and manipulating the surface wave. For a single-layer ultrathin MetaSurface, only transverse electrical currents **J**_**s**_ = **n** × **H**_**a**_ are induced (where **H**_a_ is the magnetic field on the surface, and **n** indicates the unit vector normal to the metallic area, pointing toward the half-space of interest) and symmetrically radiate on both sides of it, limiting the efficiency of the device^56^. Therefore, a single ultrathin MetaSurface is equivalent to a shunt (parallel) reactance, which can only introduce a discontinuity on the transverse magnetic field; on the other hand, the transverse electric field remains continuous. In other words, a single ultrathin MetaSurface supporting only electric currents cannot fully manipulate the wave. To determine a discontinuity of the transverse electric field, it is necessary to introduce an impedance element in series, thanks to the presence of the dielectric substrate and its finite thickness. The presence of both parallel and series impedances in the form of a П network (like our structure) can therefore determine a discontinuity in the propagating electromagnetic field, allowing full control of the wave properties.

In our MetaSurface for the single unit-cell we need to consider tangential components boundary conditions E_1tan_ = E_2tan_ and H_1tan_ = H_2tan_^57^. Therefore, the layer is equivalent to a *shunt* Impedance composed by Resistance *R*(**J**_**s**_) and a Reactance X_MS_ of inductive X_L_(**J**_**m**_) = *jωL* or capacitive X_C_(**J**_**d**_) = 1/*jωC* nature if the structure is metallic or dielectric, respectively. In presence of adjacent unit-cells, the normal components boundary conditions read B_1_ = B_2_ and D_1_ = D_2_^58^. Therefore, the adjacent structures result magnetically and/or electrically coupled. The coupling phenomena are equivalent to the mutual inductance *M*(**B**_**i**_) = L_2_/L_1_ and mutual capacitance *C*(**D**_**i**_) = C_2_/C_1_.

From the physical point of view, the subunits are arranged so close to each other, leading to a highly confined electric field at the dielectric gaps between adjacent particles. From antenna array theory^54^ we know that as the periodicity of the array elements decreases both the amplitude and phase, consequently also the bandwidth significantly changes^59^. The MetaSurface can be modeled as an array of elementary electric/magnetic dipoles (representing the unit-cells) with different dimensions and consequently operational frequencies and bandwidths. When such dipoles are far away from each other, they resonate with their own frequency like stand-alone elements. As the mutual distance among them is decreasing and such elements are getting closer each other, the entire system resonate not only at the single frequencies like before, but also new additional frequencies will rise close to the previous ones: when the optimal distance is achieved we have a constructive interference^60^, leading to a multi-band response if higher order modes are excited; and broadband structures if the single resonances are close enough each other, to create a broadband single peak.

The intra-layer coupling is always a compromise between efficiency/operational bandwidth and manufacturing capabilities: from one side, further decreasing the distance between elements will lead to a higher broadband behavior; on the other hand, it would also make the fabrication process very difficult.

## Conclusions

In this work, a new approach for the control and manipulation of electromagnetic surface-wave in terms of amplitude and phase, has been presented. The possibility to manage and tailor waves at will is achieved by using curvilinear MetaSurface structures. A new rigorous method is developed to relate the wave electromagnetic propagation characteristics with the curvilinear structure physical properties. The reliability of the proposed approach has been evaluated through modeling, design, and manufacturing for surface-wave cloaking applications. Then experimentally validated in the frequency range 8–12 GHz. Despite the structure being experimentally verified at microwave frequencies, a technique that allows the control of the mesh size when going from flat to curvilinear is useful and of great interest in many practical applications, beyond the surface-wave application. It is worth noting that by exploiting the proposed method we reduced the complexity in terms of fabrication process, to achieve feasible dimensions for both unit-cells and substrate thickness.

## Electronic supplementary material

Supplementary Information for Curvilinear MetaSurfaces for Surface Wave Manipulation

## Data Availability

All data generated or analysed during this study are included in this published article (and its Supplementary Information file).
