# Supplementary material for: Curvilinear MetaSurfaces for Surface Wave Manipulation
Source: Sci Rep. 2019 Feb 28;9:3107. doi: 10.1038/s41598-018-36451-8 (PMC6395592; doi:10.1038/s41598-018-36451-8)
Supplement: Supplementary file 1 — Supplementary Information for Curvilinear MetaSurfaces for Surface Wave Manipulation [file 41598_2018_36451_MOESM1_ESM.docx]

Supplementary Information for

**Curvilinear MetaSurfaces for Surface Wave Manipulation**

Luigi La Spada, Chris Spooner, Sajad Haq and Yang Hao

**MATERIALS AND METHODS**

**1 The wave equation derivation**

Let’s start from source free Maxwell’s Equations in the frequency domain. Let us assume that the space in which the electric/magnetic fields should be solved is source-free and lossless. The medium considered is linear, isotropic, and non-homogeneous in terms of permittivity profile ε(**r**), where ***r*** is the generic vector related to the position in a generic coordinate system:

$\left\{\begin{matrix} \nabla\times\text{E}=-j\omega\mu\text{H} \\ \nabla\times\text{H}=j\omega\varepsilon\left(\text{r}\right)\text{E} \\ \nabla\bullet\left[\varepsilon\left(\text{r}\right)\text{E}\right]=0 \\ \nabla\bullet\text{H}=0 \end{matrix}\right.$ (1)

To obtain the wave equation we need to evaluate the curl of both sides:

$\nabla\times\nabla\times\text{E}=-j\omega\mu\nabla\times\text{H}=\omega^{2}\mu\varepsilon\left(\text{r}\right)\text{E}$ (2)

The expansion of the electric field in the left side is:

$\nabla\left(\nabla\bullet\text{E}\right)-\nabla^{2}\text{E}=\omega^{2}\mu\varepsilon\left(\text{r}\right)\text{E}$ (3)

From the third Maxwell equations, it is possible to evaluate the divergence of the electric field vector:

$\nabla\bullet\left[\varepsilon\left(\text{r}\right)\text{E}\right]=\varepsilon\left(\text{r}\right)\nabla\bullet\text{E}+\nabla\varepsilon\left(\text{r}\right)\bullet\text{E}$ (4)

Leading to

$\nabla\bullet\text{E}=\text{-E}\bullet\frac{\nabla\varepsilon\left(\text{r}\right)}{\varepsilon\left(\text{r}\right)}=-\text{E}\bullet\nabla\left[\log\left(\varepsilon\left(\text{r}\right)\right)\right]$ (5)

We can now write in a complete form the desired non-homogeneous wave equation:

$\nabla^{2}\text{E}+\nabla\left[\text{E}\bullet\nabla\left[\log\left[\varepsilon\left(\text{r}\right)\right]\right]\right]+\omega^{2}\mu\varepsilon\left(\text{r}\right)\text{E}=0$ (6)

Typically, the electric field **E**(***r***, ω) is function of the generic position ***r*** and the frequency ω, but here, for simplicity we consider the electric field a scalar quantity and monochromatic. Under such conditions, eqn. (6) reduces to an ordinary linear second order differential equation, namely:

(7)

where the prime over the symbol on its right-hand side means differentiation with respect to ***r*** only. f(***r***) and g(***r***), both being some functions of ***r***, represent the pattern of non-uniformity. By denoting:

(8)

Equation (7) can be put in the form:

(9)

For arbitrary f(***r***) and g(***r***), with no inter-relationship between them, a closed formula for the general solution of eqn. (9) is not available. Consequently, solutions in closed-forms and in terms of known functions of analysis for certain special cases been given in the literature.

**
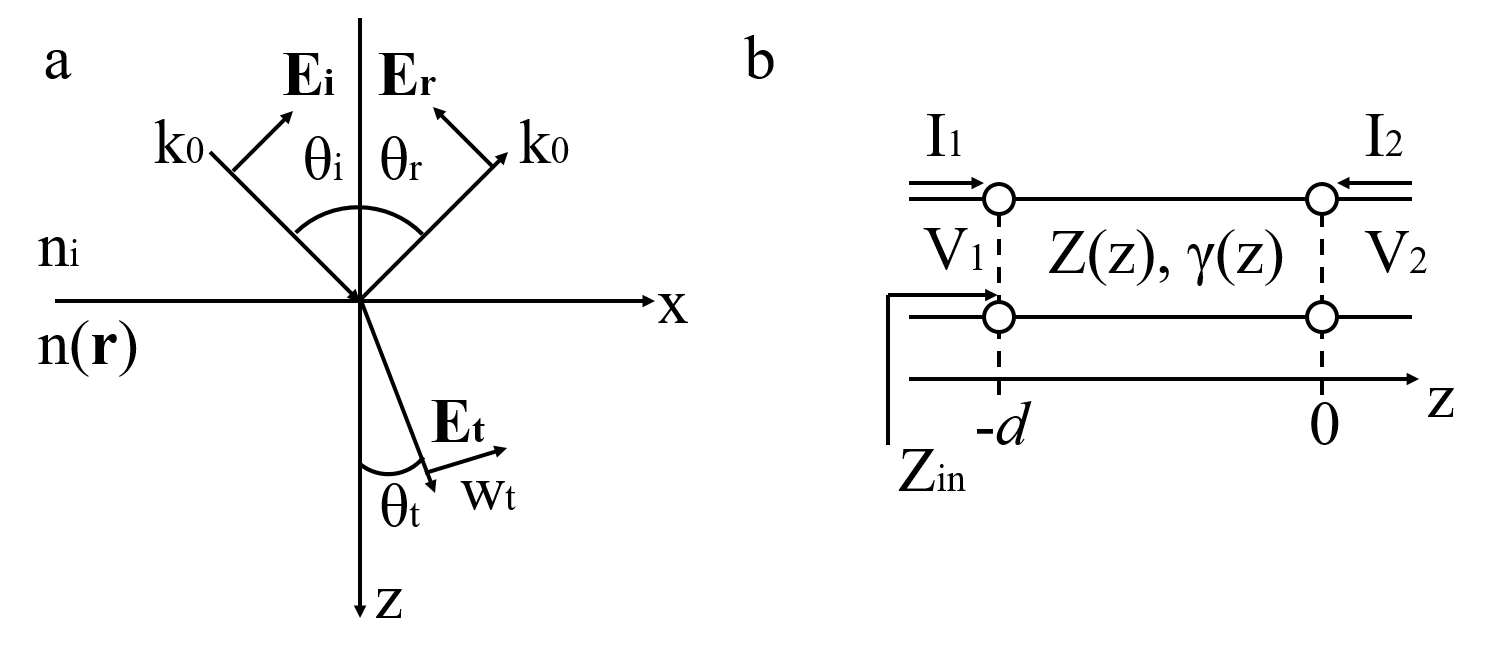
**

**Fig.1:** (a) A plane wave (linear polarization TE) impinges on an interface between a medium of homogeneous refractive index n_i_ and the other medium with non-homogeneous refractive index n(**r**); (b) The related non-homogeneous Transmission Line model

*1.1 Principle of the proposed method*

Here we develop a simple generalized method to solve eqn. (9) with solutions in closed-forms. Let’s suppose we have two half-spaces in a Cartesian coordinate system (*x, y, z*) (fig.1a), let’s assume as incidence plane, the surface (*x, y*) and the propagation direction the *z*-axis. If we know the incident wave (in the half-space *z*<0) and the medium electromagnetic characteristics at the interface (*z*=0), the transmitted wave can be obtained on the other side of the interface (in the half-space *z* > 0). To study the propagation of the new wave we need to evaluate the field on every plane *z* = cost > 0. The basic idea is to represent the field as a super-imposition of simple plane waves. For each plane wave, it will be known the propagation effect. To obtain the total field, it will be necessary only super-impose each plane wave at the desired plane *z* by using the Fourier Transform technique.

Let’s consider a single plane wave with complex amplitude: $V\left(\text{r}\right)=A\cdot e^{j\text{k}\cdot\text{r}}$. In the case of Cartesian coordinate system, the components of the wave vector **k** and vector **r,** are respectively: and . The distribution of complex amplitude evaluated at *z*=0 is of the form: $V\left(x,y,z=0\right)=A\cdot e^{j\left(k_{x}\cdot x+k_{y}\cdot y\right)}$. Therefore, the total field is simply the integral (super-imposition) of such single plane waves as follow:

$V\left(x,y,z\right)=\iint_{\text{(x,y)}} A_{z}\left(p,q\right)e^{j2\pi\left(p\cdot x+q\cdot y\right)}dpdq$ (10)

Being **(x,y)** the plane interface along we perform the integration, *p* and *q* the spatial frequency and $A_{z}\left(p,q\right)$the angular spectrum along *z*. The total field can be represented by plane waves with complex amplitude $A_{z}\left(p,q\right)$, that are homogeneous if the spatial frequencies satisfy the following relationship $p^{2}+q^{2}\leq\frac{1}{\lambda^{2}}$ and non-homogeneous otherwise. Having said that, the proposed generalized method consists in:

1) transforming eqn. (9) from ***r***-domain to some other domain by changing the independent variable ***r*** to some other new independent variable **w** = F(***r***)

2) equating the coefficients of the transformed equation with the corresponding coefficients of like-terms of a chosen standard differential equation (with well-known solution) expressed in terms of the same independent variable **w** = F(***r***).

3) From the relations, obtainable from point 2, two separate sets of expressions for f(***r***) and g(***r***) are found out.

*1.2 Mathematical formulation*

If the independent variable of ***r*** of eqn. (8) written as

(11)

is changed to a new independent variable **w** = F(***r***), then eqn. (8) reduces to:

(12)

where the dot over the symbol denotes differentiation with respect to **w** only. If the chosen standard linear second-order differential equation associated with the name of the proposed class is taken to be

(13)

with its general solution

$E=A\cdot M\left(\text{w}\right)+B\cdot N\left(\text{w}\right)$ (14)

where M(**w**) and N(**w**) are some functions of **w**, and A and B are constants to be determined from the boundary conditions of the structure and electromagnetic problem under study. Then constraining eqn. (12) to simulate eqn. (13) yields to:

(15)

Relations (15) shows that f(***r***) may be obtainable in terms of **w** which may be any arbitrary function of ***r*** or alternatively **w** can be made available in terms of arbitrary f(***r***). Corresponding to arbitrary f(***r***), the set of non-uniformities may be derived by finding out **w** in terms of f(***r***) using (15) and then expressing g(***r***) in terms of f(***r***) as given in eqn. (15). The substitution of this value of **w** in terms of arbitrary f(***r***) into solution (12) yields the general solution of electric field for the second set of non-uniformities. In our case, substitution of:

$\begin{matrix} P\left(\text{w}\right)=0 \\ Q\left(\text{w}\right)=k_{0}^{2} \end{matrix}$ (16)

Where *k* is a constant ≠ 0 reduces eq. (12) to the form of the generalized proportionate line:

(17)

having its general solution

$E=A_{1}\cdot e^{jk_{0}w}+B_{1}\cdot e^{-jk_{0}w}$ (18)

A comparison of eqns. (15) with (16) gives

(19)

After some mathematical manipulations, in terms of an arbitrary f(***r***)=ε(***r***), we have:

$w\left(\text{r}\right)=k_{1}\int\varepsilon\left(\text{r}\right)d\text{r},\text{ }g\left(\text{r}\right)=k_{1}^{2}\varepsilon\left(\text{r}\right)$ (20)

With solutions:

$E\left(\text{r}\right)=Ae^{jk_{0}\left(k_{1}\int\varepsilon\left(\text{r}\right)d\text{r}+k_{2}\right)}+Be^{-jk_{0}\left(k_{1}\int\varepsilon\left(\text{r}\right)d\text{r}+k_{2}\right)}$ (21)

*k_1_* and *k_2_* are arbitrary constants unless otherwise stated, and *k_1_* ≠ 0.

*1.3 Transmission Line model*

Now that we have reduce eqn. (9) to a simpler form of eqn. (21), also the related transmission line equations can be simplified. By using a similar procedure for the electric field in the previous paragraph, we start from the non-homogeneous telegrapher equations:

$\begin{matrix} \frac{dV\left(z\right)}{dz}=Z_{0}Z\left(z\right)V\left(z\right) \\ \frac{dI\left(z\right)}{dz}=Y_{0}Y\left(z\right)I\left(z\right) \end{matrix}$ (22)

For sake of simplicity we refer to only one dimension *z*, but everything can be done for any coordinates. The related differential equation in terms of voltage (dual for the current equation) is:

$\frac{d^{2}V\left(z\right)}{dz^{2}}-\left(\frac{1}{Z\left(z\right)}\frac{dZ\left(z\right)}{dz}\right)\frac{dV\left(z\right)}{dz}-\gamma_{0}^{2}\gamma^{2}\left(z\right)V\left(z\right)=0$ (23)

Being

$\begin{matrix} Z\left(z\right)=\sqrt{\frac{\mu_{0}}{\varepsilon_{0}}}\sqrt{\frac{\mu_{r}}{\varepsilon_{r}}}=Z_{0}\cdot f\left(z\right) \\ Y\left(z\right)=\frac{1}{Z\left(z\right)}=Y_{0}\cdot g\left(z\right) \\ \gamma^{2}\left(z\right)=\sqrt{\mu_{0}\varepsilon_{0}}Z\left(z\right)Y\left(z\right)=\gamma_{0}\cdot f\left(z\right)\cdot g\left(z\right) \end{matrix}$ (24)

By using the technique detailed in the previous paragraph (1.2), we obtain the related traveling wave solutions as

$\left\{\begin{matrix} V\left(z\right)=V_{0}^{+}e^{\Phi\left(z\right)}+V_{0}^{-}e^{-\Phi\left(z\right)} \\ I\left(z\right)=\frac{V_{0}^{+}}{Z_{0}}e^{\Phi\left(z\right)}-\frac{V_{0}^{-}}{Z_{0}}e^{-\Phi\left(z\right)} \end{matrix}\right.$ (25)

With $\Phi\left(z\right)=\gamma_{0}\int Z\left(z\right)dz+\phi$

where the e^−Ф(z)^ term represents wave propagation in the +z direction, and the e^+ Ф(z)^ term represents wave propagation in the −z direction.

Figure 1(b) shows a lossless non-homogeneous transmission line terminated in an arbitrary load impedance Z_L_. Assume that an incident wave of the form V^+^_0_ e^−jβz^ is generated from a source at z < 0. We know that the ratio of voltage to current for such a traveling wave is Z_0_, the characteristic impedance of the line. However, when the line is terminated in an arbitraryload Z_L_ ≠ Z_0_, the ratio of voltage to current at the load must be Z_L_. Thus, a reflected wave must be excited with the appropriate amplitude to satisfy this condition.

The total voltage and current at the load are related by the load impedance, so at z = 0 are:

$\left\{\begin{matrix} V_{2}=V\left(0\right)=V_{0}^{+}e^{-\Phi\left(0\right)}+V_{0}^{-}e^{\Phi\left(0\right)} \\ -I_{2}=I\left(0\right)=\frac{V_{0}^{+}}{Z_{c}\left(0\right)}e^{-\Phi\left(0\right)}-\frac{V_{0}^{-}}{Z_{c}\left(0\right)}e^{\Phi\left(0\right)} \end{matrix}\right.$ (26)

From which we have

$\left\{\begin{matrix} V_{0}^{+}=\frac{V_{2}-Z\left(0\right)I_{2}}{2}e^{^{+\Phi\left(0\right)}} \\ V_{0}^{-}=\frac{V_{2}+Z\left(0\right)I_{2}}{2}e^{^{-\Phi\left(0\right)}} \end{matrix}\right.$ (27)

The total voltage and current at the input of the line, so at z=-d are:

$\left\{\begin{matrix} V_{1}=V\left(d\right)=V_{0}^{+}e^{-\Phi\left(d\right)}+V_{0}^{-}e^{+\Phi\left(d\right)} \\ I_{1}=I\left(d\right)=\frac{V_{0}^{+}}{Z_{c}\left(d\right)}e^{-\Phi\left(d\right)}-\frac{V_{0}^{-}}{Z_{c}\left(d\right)}e^{+\Phi\left(d\right)} \end{matrix}\right.$ (28)

We write V_1_ and I_1_ as a function of V_2_ and I_2_:

$\left\{\begin{matrix} V_{1}=V_{2}\cosh\left[\Phi\left(0\right)-\Phi\left(d\right)\right]-Z_{c}\left(0\right)I_{2}\sinh\left[\Phi\left(0\right)-\Phi\left(d\right)\right] \\ I_{1}=\frac{V_{2}}{Z_{c}\left(d\right)}\sinh\left[\Phi\left(0\right)-\Phi\left(d\right)\right]-\frac{Z_{c}\left(0\right)}{Z_{c}\left(d\right)}I_{2}\cosh\left[\Phi\left(0\right)-\Phi\left(d\right)\right] \end{matrix}\right.$ (29)

The input impedance will be:

$Z_{in}\left(z\right)=Z_{0}\frac{Z_{L}\cosh\left(k_{0}L\right)+Z_{0}\sinh\left(k_{0}L\right)}{Z_{L}\sinh\left(k_{0}L\right)+Z_{0}\cosh\left(k_{0}L\right)}$ (30)

With $L=\left.\Phi\left(z\right)\right|_{0}^{d}=\int_{0}^{d} Z\left(z\right)dz$

And the related reflection coefficient is: $r=\left|\frac{Z_{in}\left(z\right)-Z_{0}}{Z_{in}\left(z\right)+Z_{0}}\right|^{2}$

This is an important result giving the input impedance of a length of transmission line with

an arbitrary load impedance. We will refer to this result as the non-homogeneous transmission line impedance equation.

**2) Substrate electromagnetic characterization**

The substrate sample was characterized in terms of permittivity by using a Split Cylinder Cavity Resonator approach (Keysight 85072A) at 10 GHz. The resonator measures the relative complex permittivity (real and imaginary part) and loss tangent of sheet materials. The Vector Network Analyser (Agilent N5230C), cables and software, complete the test system. The split cylinder resonator is a cylindrical resonant cavity separated into two halves. The sample is loaded in a gap between the two-cylinder halves. One-cylinder half is fixed, and the other adjusts allowing the gap to accommodate varying sample thicknesses. Using the in-built machine “mode matching” model, permittivity and loss tangent is calculated at the TE_011_ mode as well as higher order modes. The sample under study, is assumed to be non-magnetic loss less ($\mu_{r}=1-j0$), homogeneous and isotropic. Values for thickness samples are in the range 0.2 – 1.5 mm.

**3) Analytical – numerical - experimental model comparison**


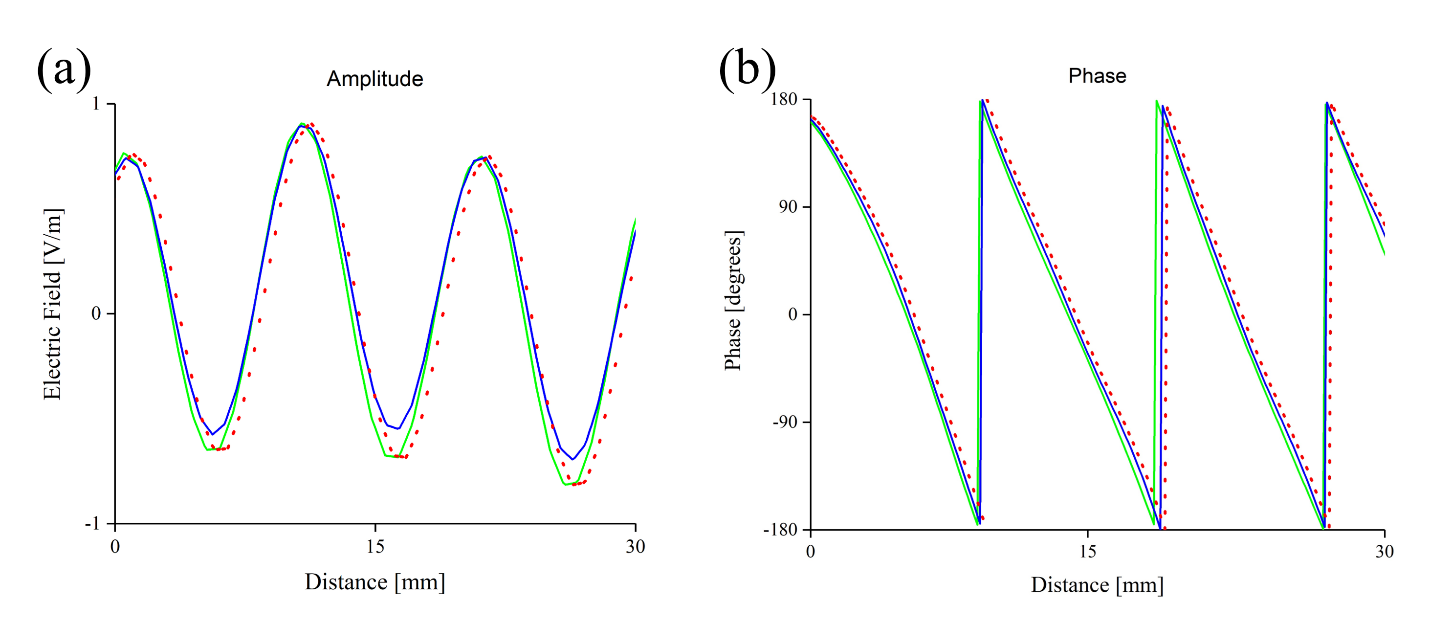


Fig.S1: Analytical (red) Numerical (blue) and Experimental (green) models’ comparison in terms of amplitude and phase at 10GHz along a probe line length 30mm positioned after the object to cloak for the MetaSurface cloak sample.
